# Homœopathic Therapeutics of Diarrhœa, Etc.

**Published:** 1881-08

**Authors:** 


### BOOK NOTICES AND REVIEWS.

---

THE HOMŒOPATHIC THERAPEUTICS OF DIARRHŒA, CHOLERA, CHOLERA MORBUS, CHOLERA INFANTUM, and all Other Loose Evacuations of the Bowels. By *James B. Bell, M. D.* Second Edition, revised and enlarged, by Drs. J. B. Bell and W. T. Laird. pp. 271, cloth, price $1.50. Bœricke & Tafel: New York and Philadelphia. London: Trübner & Co., Ludgate Hill. Homœopathic Publishing Co., 2 Finsbury Circus. 1881.

This little book, issued in 1869, by Dr. Bell, has long been a standard work in Homœopathic Therapeutics. We feel quite within bounds in asserting that it has been the means, under our law, of saving thousands of lives. Than this, no greater commendation could be penned.

In this second edition, Dr. Bell has been assisted by his late partner, Dr. Laird, of Maine; also by Drs. Lippe, William P. Wesselhoeft and E. A. Farrington. Thirty-eight new remedies are given; the old text largely re-written; many rubrics added to the repertory; a new feature, the "black type," for especially characteristic symptoms, introduced.

This is a typical homœopathic work, which no homœopathic physician can afford to be without.

The typographical setting is worthy of the book.

